# An evaluation of circulating activated TAFI in septic DIC: a case series and review of the literature

**DOI:** 10.1186/s12959-022-00364-4

**Published:** 2022-02-07

**Authors:** Takaaki Totoki, Takashi Ito, Midori Kakuuchi, Nozomi Yashima, Ikuro Maruyama, Yasuyuki Kakihana

**Affiliations:** 1 Department of Anesthesiology & Critical Care Medicine, Kyushu University, Fukuoka, Japan; 2 Department of Emergency and Intensive Care Medicine, Kagoshima University Graduate School of Medical and Dental Sciences, Kagoshima, Japan; 3 Department of Systems Biology in Thromboregulation, Kagoshima University Graduate School of Medical and Dental Sciences, Kagoshima, Japan

**Keywords:** Disseminated intravascular coagulation, Thrombin-activatable fibrinolysis inhibitor, Sepsis, Thrombomodulin

## Abstract

**Background:**

Administration of recombinant human soluble thrombomodulin (rTM) is often used in Japan to treat septic disseminated intravascular coagulation (DIC). Thrombin-activatable fibrinolysis inhibitor (TAFI) is a fibrinolysis inhibitor activated by the thrombin-thrombomodulin complex, however, it is unknown whether circulating activated TAFI is increased after rTM administration in patients with DIC. Furthermore, the relationship between TAFI activation and the prognosis of septic DIC is not defined yet.

**Case presentation:**

We report a series of 8 patient’s TAFI activation with septic DIC treated by rTM. We sought to investigate the effect of rTM on TAFI activation and the association of plasma activated TAFI (TAFIa/ai) levels with the prognosis of septic DIC.

Using plasma samples from clinical studies conducted from May 2016–March 2017 on eight patients with septic DIC at Kagoshima University Hospital, we measured plasma levels of total TAFI, TAFIa/ai, thrombin-antithrombin complex (TAT), prothrombin fragment 1 + 2 (F1 + 2), soluble fibrin (SF), antithrombin (AT), protein C (PC), protein S (PS), and plasminogen activator inhibitor-1 (PAI-1) before and after intravenous rTM administration. Then, we evaluated the relationship of these marker levels to prognosis. The thrombin-rTM complex activated TAFI in vitro in plasma from a healthy volunteer. However, TAFIa/ai levels did not significantly increase over baseline in the septic DIC patients after intravenous rTM administration. Baseline TAFIa/ai levels in non-survivors were significantly higher than those in survivors.

**Conclusions:**

Plasma TAFIa/ai did not increase with rTM administration. Elevated baseline TAFIa/ai concentration may be a negative prognostic indicator in septic DIC. Larger studies are needed to confirm the in vivo effect of rTM on TAFI activation.

## Introduction

Thrombin-activatable fibrinolysis inhibitor (TAFI) is synthesized and secreted by the liver [1]. TAFI can be activated by thrombin, and this reaction is markedly promoted by thrombomodulin, an anticoagulant protein expressed on the surface of endothelial cells [2]. During fibrinolysis, plasmin partially hydrolyzes fibrin, and plasminogen, plasmin, and tissue-type plasminogen activator (tPA) bind to the C terminal lysine residue generated in partially-hydrolyzed fibrin. Activated TAFI inhibits the binding of plasminogen, plasmin, and tPA by selectively excising the lysine residue at the C-terminal of fibrin, thereby suppressing the fibrinolytic reaction and controlling the rate of fibrinolysis [3–5].

In sepsis, the expression of endothelial anticoagulant proteins, including thrombomodulin, is suppressed by endothelial damage. The decrease in thrombomodulin expression in the endothelium results in a hypercoagulable state and sepsis-associated disseminated intravascular coagulation (DIC) [6]. In Japan, recombinant human soluble thrombomodulin (rTM) is commonly used in expectation of activated protein C (APC)-dependent anticoagulant effects to counteract the hypercoagulable state in septic DIC [7]. Theoretically, administration of rTM may not only promote thrombin-mediated protein C activation but also promote thrombin-mediated TAFI activation, however, activated TAFI levels after rTM administration is unknown. Furthermore, the relationship between TAFI activation and the prognosis of septic DIC is not defined yet. In this study, we analyzed activated TAFI levels after rTM administration in patients with septic DIC.

The activated TAFI levels in no-survivors tended to be higher than in survivors.

## Case presentation

We have treated eight patients with septic DIC in our prior research on activated protein C conducted May 2016–March 2017 in Kagoshima University Hospital [8].

The study was conducted using their plasma samples. This prospective observational study conformed to the provisions of the Declaration of Helsinki and was approved by the Ethics Committee of Kagoshima University Hospital. Between May 2016–March 2017, written informed consent was obtained from eight patients with sepsis-associated DIC prior to participation.

The diagnosis of sepsis and DIC was made according to the Third International Consensus Definition for Sepsis (Sepsis-3) [9] and the diagnostic criteria established by the Japanese Association for Acute Medicine (JAAM DIC criteria) [10], respectively.

### Sample preparation for TAFI assays

Plasma from a healthy volunteer was incubated for 10 min at 37 °C with varying concentrations of human thrombin (Sigma-Aldrich, St. Louis, MO, USA) and rTM (Asahi Kasei Pharma, Tokyo, Japan). The reaction was terminated by addition of a protease inhibitor cocktail containing hirudin (Sekisui Medical, Tokyo, Japan). The samples were centrifuged at 4 °C and the supernatants were stored at − 80 °C until analysis of TAFI concentrations.

Blood samples collected from the eight patients with sepsis-associated DIC before and after administration of rTM (Asahi Kasei Pharma, Tokyo, Japan), (130 or 380 U/kg, depending on renal function) via intravenous drip infusion on day 1 and day 2 were immediately anticoagulated with a one-tenth volume of sodium citrate and kept at 4 °C. The samples were then centrifuged at 2000×g for 10 min at 4 °C and plasma samples were stored at − 80 °C until analysis of TAFI concentrations.

### Measurement of plasma levels of total TAFI, activated TAFI, TAT, F1 + 2, SF, AT, PC, PS, sTM, and PAI-1

Plasma total TAFI levels were analyzed by the Imuclone Total TAFI ELISA (Sekisui Diagnostics, Stamford CT, USA). Plasma activated TAFI levels (TAFIa/ai) were analyzed by the Asserachrom TAFIa/TAFIai (Diagnostica Stago, Seine, France). Plasma thrombin-antithrombin complex (TAT) levels were analyzed using Stacia chemiluminescence enzyme immunoassay (CLEIA) TAT (LSI Medience, Tokyo, Japan). Plasma prothrombin fragment 1 + 2 (F1 + 2) levels were analyzed using Enzygnost F1 + 2 (Siemens Healthcare Diagnostics, Tokyo, Japan). Plasma soluble fibrin (SF) levels were analyzed using Iatro SF II (LSI Medience). Antithrombin (AT) was analyzed by a synthetic chromogenic substrate method using HemosIL ATLQ (Instrumentation Laboratory, Bedford MA, USA). Plasma protein C (PC) levels were measured by a synthetic chromogenic substrate method using HemosIL Protein C (Instrumentation Laboratory). Plasma protein S (PS) levels were measured by a synthetic chromogenic substrate method using HemosIL PS clot (Instrumentation Laboratory). Plasminogen activator inhibitor-1 (PAI-1) was analyzed using an LPIA • tPAI test (LSI Medience). All of the preceding assays were performed according to the manufacturers’ instructions.

## Discussion and conclusions

**Fig. 1:**
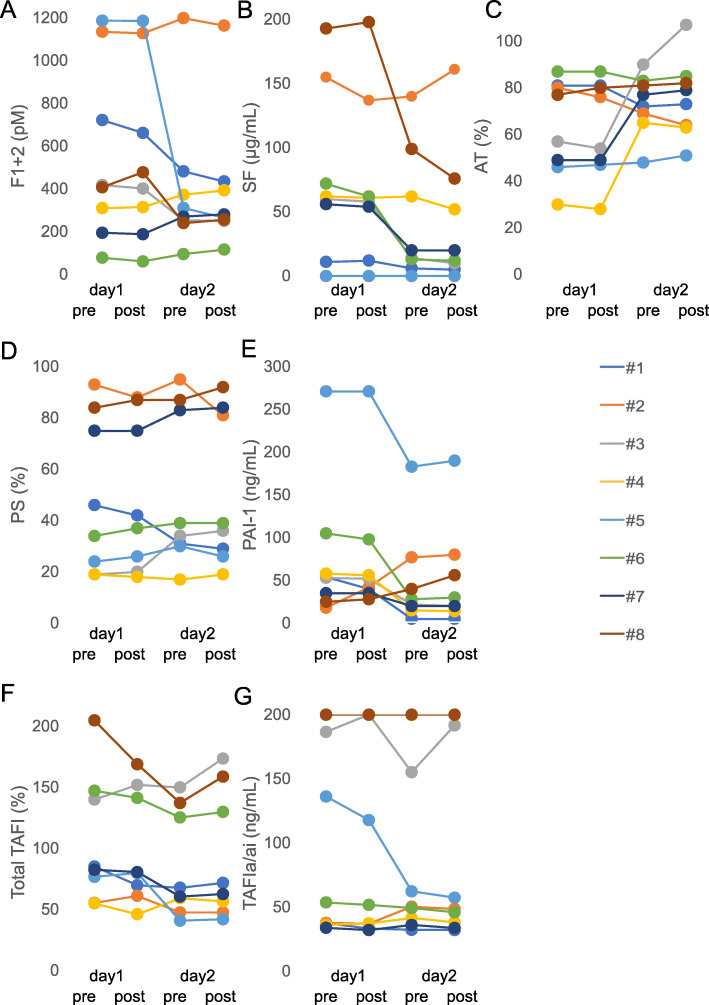
Changes in coagulation/fibrinolysis markers in patients with sepsis-associated DIC before and after rTM administration. Blood samples were collected from the eight patients with sepsis-associated DIC before and after administration of rTM on day 1 and day 2. (A) Prothrombin fragment 1 + 2 (F1 + 2), (B) soluble fibrin (SF), (C) antithrombin (AT), (D) protein S (PS), (E) plasminogen activator inhibitor-1 (PAI-1), (F) total thrombin-activatable fibrinolysis inhibitor (TAFI), and (F) activated TAFI (TAFIa/ai) levels were analyzed according to the manufacturers’ instructions. TAFIa/ai levels were not increased after rTM treatment except for the case #3 and #8. In the case of #8, TAFIa/ai levels at any points were higher than 200 ng/mL, the upper limit of this measurement, however, the absorbance values were increased after rTM treatment from 2.19 to 2.32 on day 1, and from 2.48 to 2.62 on day 2

We examined the plasma levels of total TAFI, TAFIa/ai, F1 + 2, SF, AT, PS, and PAI-1 in eight patients with sepsis-associated DIC before and after administration of rTM over 2 days (Fig. 1).

The background characteristics of the eight patients are shown in Supplementary Table 1. Plasma soluble TM levels rapidly increased during a 30–60-min period of rTM administration (380 U/kg except for cases #3 and #5) to reach around 1 μg/mL [8]. In these conditions, plasma TAFIa/ai levels were not increased after rTM treatment except for the cases #3 and #8.

In this study, we found that thrombin-rTM did not promoted increase plasma TAFIa/ai levels in most patients with sepsis-associated DIC. Three (#3, #6, and #8) out of eight patients showed high total TAFI levels before rTM administration, and two (#3 and #8) out of the three patients showed high thrombin generation as evidenced by high F1 + 2 values. TAFIa/ai levels were increased in these two patients (#3 and #8) after rTM administration, suggesting that TAFIa/ai levels could be increased after rTM administration in patients with high total TAFI levels and high thrombin generation. However, this hypothesis is based on the data of eight patients, and thus larger scale analysis is necessary to confirm it.

Extensive activation of TAFI has been reported as an independent predictor of mortality in sepsis [11]. Our findings support this hypothesis in patients with sepsis-associated DIC. Increased TAFIa/ai may cause ischemic organ failure by inhibiting intravascular fibrinolysis. Increased TAFIa/ai may be the result of increased thrombin generation, which is also thought to be associated with ischemic organ failure and poor outcome. So, extensive activation of TAFI may be the cause or the result of critically ill conditions. In the latter case, rTM administration can improve outcome by suppressing thrombin generation. In the former case, rTM administration may not worsen outcome because rTM administration did not increase activated TAFI levels in most cases. However, it should be noted that activated TAFI levels can be increased after administration of rTM if baseline levels of total TAFI and thrombin generation are simultaneously elevated. Previous studies showed that inhibition or knockout of TAFI alleviated sepsis-induced organ injury in mice [12, 13], indicating that increased TAFIa/ai may account at least in part for sepsis-induced organ failure.

Plasma TAFIa/ai did not increase with rTM administration. The activated TAFI levels in no-survivors tended to be higher than in survivors. Therefore elevated baseline TAFIa/ai concentration may be a negative prognostic indicator in septic DIC. Larger studies are needed to confirm the in vivo effect of rTM on TAFI activation.

## Data Availability

The datasets used and/or analyzed during the current study are available from the corresponding author on reasonable request.

## Abbreviations

AT: antithrombin
DIC: Disseminated intravascular coagulation
ELISA: Enzyme-linked immunosorbent assay
FDP: Fibrin/fibrinogen degradation products
F1 + 2: Prothrombin fragment 1 + 2
PAI-1: plasminogen activator inhibitor-1
rTM: Recombinant human soluble thrombomodulin
SF: soluble fibrin
sTM: soluble thrombomodulin
TAFI: thrombin-activatable fibrinolysis inhibitor
TAFIa: Activated thrombin activatable fibrinolysis Inhibitor
TAT: Thrombin-antithrombin complex
